# Ferroptosis, lipid metabolism, and genetic regulation in postoperative rehabilitation of elderly hip fractures: from molecular mechanisms to clinical translation

**DOI:** 10.3389/fgene.2026.1852662

**Published:** 2026-06-17

**Authors:** Xiaojun Xie, Yuxia Zhu, Xuan Lu, Yingui Guo, Shuyang Xiao, Xin Li, Huishan Lu, Ganqing Li, Huishi Liu

**Affiliations:** 1 Second Department of Orthopedics, Dongguan Hospital of Traditional Chinese Medicine, Dongguan, Guangdong, China; 2 Department of Nursing, Dongguan Hospital of Traditional Chinese Medicine, Dongguan, Guangdong, China; 3 The Fifth Department of Orthopedics, Dongguan Hospital of Traditional Chinese Medicine, Dongguan, Guangdong, China; 4 The Fourth Department of Orthopedics, Dongguan Hospital of Traditional Chinese Medicine, Dongguan, Guangdong, China

**Keywords:** elderly, ferroptosis, hip fracture, pathological fracture, postoperative rehabilitation, tumor bone metastasis

## Abstract

**Background:**

Elderly hip fracture represents a major global public health challenge, with postoperative rehabilitation involving complex molecular mechanisms and metabolic networks. Beyond osteoporotic fractures, pathological hip fractures caused by tumor bone metastasis constitute a non-negligible proportion in elderly patients, with molecular pathological mechanisms significantly different from osteoporotic fractures. Recent research has confirmed that ferroptosis (iron-dependent regulated cell death) and lipid metabolism dysregulation are key links in fracture healing and functional recovery, while genetic polymorphisms, epigenetic modifications, and tumor microenvironment modulate these processes through multiple pathways. Nursing assessment and intervention play an irreplaceable role in promoting functional recovery and preventing complications.

**Objective:**

This review systematically elucidates the molecular mechanisms of ferroptosis and lipid metabolism in postoperative rehabilitation of elderly hip fractures (including tumor metastatic pathological fractures), explores the genetic and epigenetic regulatory networks, analyzes multidisciplinary nursing intervention strategies, and provides a theoretical basis for translating basic research into clinical application.

**Methods:**

PubMed, Web of Science, Embase, and CNKI were searched to include basic and clinical research published from 2015 to 2025. Keywords included ferroptosis, lipid metabolism, hip fracture, tumor bone metastasis, pathological fracture, genetic regulation, nursing intervention, and muscle atrophy.

**Results:**

Ferroptosis participates in secondary injury and inflammatory responses at fracture sites through lipid peroxidation, glutathione depletion, and iron overload. Lipid metabolism dysregulation, especially polyunsaturated fatty acid (PUFA) metabolic abnormalities, not only promotes ferroptosis but also affects bone remodeling and muscle metabolism. Tumor bone metastasis exacerbates ferroptosis after pathological fractures by disrupting local iron metabolism homeostasis, upregulating ACSL4 expression, and promoting oxidative stress. Genetic polymorphisms of key regulatory factors such as GPX4 and FSP1, and DNMT-mediated epigenetic silencing, are closely related to prognosis in elderly patients. Systematic nursing interventions including nutritional management, pain control, early mobilization, and psychological support improve functional outcomes by regulating the oxidative stress-inflammation axis.

**Conclusion:**

Ferroptosis, lipid metabolism, genetic regulation, and nursing interventions together constitute a complex regulatory network in postoperative rehabilitation of elderly hip fractures (including pathological fractures), providing multi-level clinical intervention targets. Future research should focus on establishing elderly- and tumor-specific fracture assessment systems, developing highly selective ferroptosis-regulating drugs, advancing clinical translation of multi-omics biomarkers, and building precision multidisciplinary rehabilitation programs integrating nursing care.

## 1 Introduction: clinical challenges and molecular basis

### 1.1 Epidemiology and clinical burden of elderly hip fractures

The global aging process continues to accelerate, with the population aged 60 and above expected to reach 2.1 billion by 2050, doubling from 2020 (Wu et al., 2021). Hip fracture is the most severe type of osteoporotic fracture in the elderly, with incidence increasing significantly with age (Sing et al., 2023; Haleem et al., 2023). In China, the population over 60 years exceeds 280 million, with an annual hip fracture incidence of approximately 250–300 per 100,000 persons, showing a continuous upward trend. Hip fractures not only pose an immediate life-threatening risk but also lead to long-term functional disability: 1-year mortality after injury is approximately 20%–30%, and among survivors, only 40%–60% can return to pre-fracture functional levels, with over 50% requiring long-term care support (Viganò et al., 2023).

The etiology of elderly hip fractures is heterogeneous. In addition to low-energy fall fractures caused by osteoporosis, pathological fractures caused by tumor bone metastasis constitute a non-negligible subtype. Malignancies such as lung cancer, breast cancer, prostate cancer, renal cancer, and multiple myeloma are highly prone to bone metastasis, with the proximal femur being a common metastatic site due to its abundant red marrow blood supply (Jiang et al., 2024). Studies show that approximately 8%–15% of hip fractures in elderly tumor patients are pathological fractures. These patients face more complex postoperative rehabilitation challenges due to tumor microenvironment effects, immunosuppression, and damage from prior chemotherapy and radiotherapy (Macedo et al., 2017; Liu et al., 2026). Pathological fracture patients exhibit more extensive local bone destruction, more intense inflammatory responses, and particularly prominent iron metabolism dysregulation and lipid peroxidation injury; understanding their unique molecular mechanisms is crucial for developing precise rehabilitation strategies.

The complexity of postoperative rehabilitation in elderly hip fractures stems from multiple overlapping pathophysiological changes: local traumatic response of the fracture itself, systemic inflammatory state induced by surgical stress, muscle atrophy and cardiopulmonary functional decline caused by bed rest, and inherent metabolic dysfunction and multimorbidity in elderly individuals (Handoll et al., 2021; Yan et al., 2022). These factors interact to form vicious cycles that severely impede rehabilitation. Traditional rehabilitation strategies have focused primarily on physical training and functional exercise, with insufficient understanding of the molecular mechanisms during the rehabilitation process, limiting the precision and effectiveness of interventions (Fairhall et al., 2022; Lewis et al., 2022).

### 1.2 Ferroptosis and lipid metabolism: an emerging molecular regulatory axis

**FIGURE 1:**
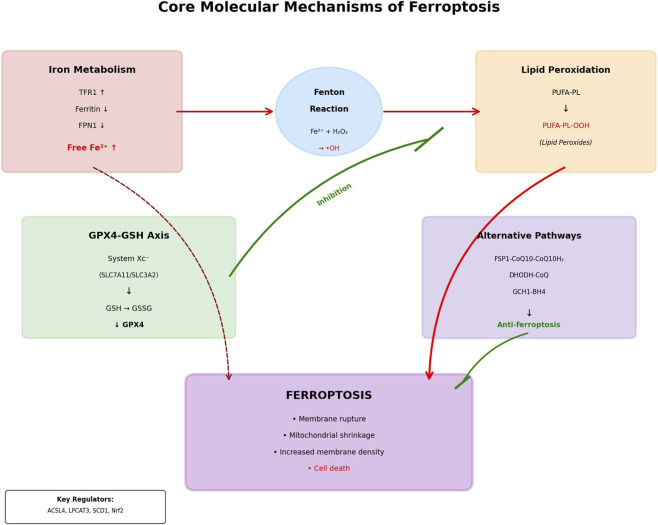
Core Molecular Mechanisms of Ferroptosis. The diagram illustrates key pathways including iron metabolism, Fenton reaction, lipid peroxidation, and the GPX4-GSH axis. Alternative pathways (FSP1-CoQ10, DHODH, GCH1-BH4) provide additional protection against ferroptosis. Green arrows indicate inhibitory (protective) effects; red pathway indicates promotion of ferroptotic cell death.

Ferroptosis was first named by Dixon et al., in 2012 as a special form of regulated cell death distinct from apoptosis, necrosis, and autophagy (Jiang et al., 2021; Tang et al., 2021). Its core features include iron-dependent accumulation of lipid peroxides, mitochondrial morphological changes (reduced volume, fewer cristae, increased membrane density), and loss of cell membrane integrity (Stockwell, 2022; Yan et al., 2021). Ferroptosis does not depend on caspase activation but is driven by excess iron ions catalyzing reactive oxygen species (ROS) generation, leading to peroxidation of PUFA-containing phospholipids, ultimately causing membrane damage and cell death (Zheng and Conrad, 2020; Berndt et al., 2024) (Figure 1).

Lipid metabolism and ferroptosis are closely linked, forming a complex regulatory network (Liang et al., 2022; Pope and Dixon, 2023). Lipids are not only structural components of cell membranes but also key molecules for energy storage, signal transduction, and metabolic regulation. Multiple levels of lipid metabolism participate in ferroptosis regulation: the synthesis and remodeling of PUFAs determine sensitivity to lipid peroxidation (Doll et al., 2017; Kagan et al., 2017); lipid peroxidation products (such as 4-HNE and MDA) serve as signaling molecules that amplify oxidative stress (Rochette et al., 2023; Ayala et al., 2014); and the antioxidant enzyme system (especially GPX4) provides protection by reducing lipid peroxides (Liu et al., 2023). Decreased lipid metabolism function and increased ferroptosis sensitivity in elderly individuals may jointly promote adverse outcomes after fractures (López-Otín et al., 2023).

At the genetic and epigenetic levels, GPX4 gene variants, ACSL4 promoter methylation, iron metabolism-related miRNA regulatory networks, and other factors vary significantly between individuals, directly affecting ferroptosis susceptibility and rehabilitation trajectories (Pei et al., 2022). Understanding these gene-environment-epigenetics interactions is the theoretical premise for achieving precision rehabilitation.

**FIGURE 2:**
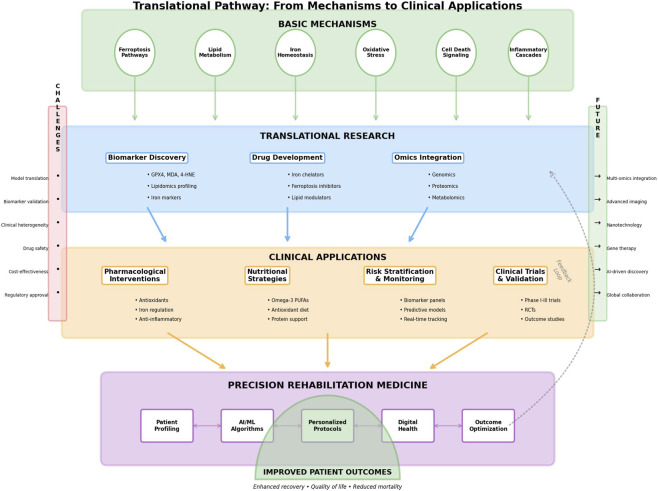
Translational Pathway from Mechanisms to Clinical Applications. The diagram shows the progression from basic mechanisms through translational research and clinical applications to precision rehabilitation medicine. A feedback loop represents continuous optimization from clinical outcomes to basic research. The ultimate goal is improved patient outcomes, better recovery quality, quality of life, and reduced mortality.

**FIGURE 3:**
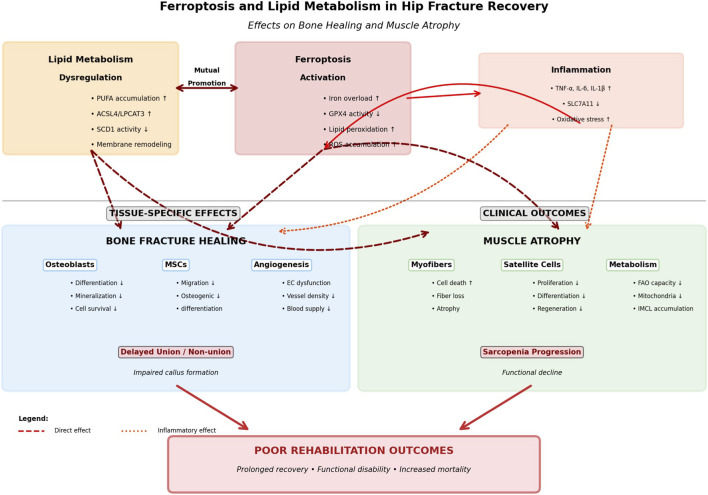
Effects of Ferroptosis and Lipid Metabolism on Hip Fracture Recovery. The upper section shows the mutually promoting relationship between lipid metabolism dysregulation and ferroptosis activation, with inflammation serving as an amplifier. The lower section shows tissue-specific effects on fracture healing (left) and muscle atrophy (right), ultimately leading to poor rehabilitation outcomes.

Recent studies indicate that ferroptosis and lipid metabolism abnormalities play important roles in various age-related degenerative diseases (such as Alzheimer’s disease, cardiovascular disease, and sarcopenia) (Berndt et al., 2024; Fang et al., 2023; Huang et al., 2021). Notably, ferroptosis has also been implicated in perioperative organ injury, including sepsis-induced myocardial damage, underscoring its clinical relevance across surgical and critical care contexts (Wang et al., 2025). In the musculoskeletal system, dysregulation of these processes is closely associated with bone loss, muscle atrophy, and functional decline (Ru et al., 2023; Cruz-Jentoft et al., 2019). However, the specific mechanisms, interaction patterns, genetic regulatory networks, and nursing intervention value of ferroptosis and lipid metabolism in postoperative rehabilitation of elderly hip fractures (including pathological fractures) have not been systematically elucidated; this review aims to fill this gap (Figures 2, 3).

## 2 Molecular mechanisms of ferroptosis and its role in the musculoskeletal system

### 2.1 Core regulatory pathways of ferroptosis

#### 2.1.1 Iron metabolism and ROS generation

Iron is an essential trace element for normal cellular functions, participating in DNA synthesis, oxygen transport, energy metabolism, and many other physiological processes (Chen et al., 2020). However, free iron (Fe^2+^) can catalyze the generation of highly reactive hydroxyl radicals (·OH) through Fenton and Haber-Weiss reactions, triggering chain reactions of lipid peroxidation (Jiang et al., 2021; Fang et al., 2023; Wei et al., 2025). Cellular iron homeostasis is precisely regulated by iron uptake (mediated by transferrin receptor TFR1), storage (regulated by ferritin), and export (membrane iron transporter FPN1) (Chen et al., 2020; Brissot et al., 2020). Elderly individuals exhibit decreased iron metabolism regulatory function, manifesting as reduced ferritin expression and weakened iron export capacity, leading to intracellular free iron accumulation (López-Otín et al., 2023; Sies et al., 2022).

After fracture trauma, local tissue damage triggers hemoglobin release and heme degradation, further increasing iron burden (Einhorn and Gerstenfeld, 2015; Salhotra et al., 2020). Heme oxygenase-1 (HO-1) catalyzes heme degradation into free iron, carbon monoxide, and biliverdin (Chang et al., 2018). Although HO-1 has a protective role in antioxidant stress, excessive activation can lead to iron overload (Dodson et al., 2019; Anandhan et al., 2020). Studies show that serum ferritin levels are significantly elevated in hip fracture patients and are associated with increased risk of postoperative complications and poor functional recovery (Haleem et al., 2023; Yan et al., 2022).

In tumor pathological fractures, the high iron demand of tumor cells (“iron addiction” phenomenon) combined with iron release at the fracture site causes more severe iron metabolism imbalance. Tumor cells highly express TFR1 to take up iron while downregulating FPN1 to impede iron export, creating local iron overload in the tumor microenvironment that provides the material basis for excessive ferroptosis activation; however, tumor cells simultaneously escape ferroptosis by upregulating GPX4 and other mechanisms, and this paradoxical state is particularly prominent in bone metastasis lesions (Coleman et al., 2020; Bebber et al., 2020).

#### 2.1.2 Lipid peroxidation and membrane damage

Lipid peroxidation is the hallmark event of ferroptosis (Jiang et al., 2021; Stockwell, 2022). Cell membranes are rich in PUFAs, especially arachidonic acid (AA) and adrenic acid (AdA), whose double bond structures are highly sensitive to oxidative damage (Doll et al., 2017; Kagan et al., 2017). The lipid peroxidation process occurs in three stages: in the initiation stage, ROS attack PUFA carbon-hydrogen bonds to generate lipid radicals (L·); in the propagation stage, lipid radicals react with oxygen to generate peroxy radicals (LOO·), which attack adjacent PUFAs to produce lipid peroxides (LOOH) and new lipid radicals, forming a chain reaction; in the termination stage, antioxidants scavenge free radicals (Zheng and Conrad, 2020; Rochette et al., 2023).

Lipid peroxidation leads to decreased membrane fluidity, dysfunction of membrane proteins, and membrane pore formation, ultimately causing cell death (Liang et al., 2022). Lipid peroxidation products such as 4-hydroxynonenal (4-HNE) and malondialdehyde (MDA) are not only damage markers but also biologically active, capable of modifying proteins and DNA and amplifying oxidative stress signals (Ayala et al., 2014). During fracture healing, excessive lipid peroxidation damages osteoblasts, chondrocytes, and vascular endothelial cells, delaying callus formation and vascular regeneration (Zhang et al., 2023a; Zhao et al., 2022; Bi et al., 2023).

#### 2.1.3 GPX4-glutathione system: central inhibitory pathway of ferroptosis

Glutathione peroxidase 4 (GPX4) is the key defense enzyme against ferroptosis, reducing lipid peroxides (LOOH) to the corresponding alcohols (LOH) to block the lipid peroxidation chain reaction (Xie et al., 2023). GPX4 activity depends on reduced glutathione (GSH) as an electron donor, while GSH is generated from cystine taken up by system xc^−^ (composed of SLC7A11 and SLC3A2) (Tang et al., 2021; Lei et al., 2022). Therefore, the GPX4-GSH-system xc^−^ axis constitutes the core axis of ferroptosis inhibition (Jiang et al., 2021; Stockwell, 2022).

In the elderly population, GPX4 expression and activity are universally decreased, associated with increased oxidative stress and the occurrence of various age-related diseases (López-Otín et al., 2023). Studies show that GPX4 expression at the fracture site in elderly hip fracture patients is significantly lower than in younger patients, associated with increased risk of delayed fracture healing and non-union (Zhang et al., 2023a; Ruan et al., 2024). System xc^−^ dysfunction leads to insufficient cystine uptake, further weakening GPX4 antioxidant capacity. Inflammatory cytokines (such as TNF-α and IL-1β) can downregulate SLC7A11 expression, forming a positive feedback loop of inflammation-oxidative stress-ferroptosis (Tang et al., 2021; Chen et al., 2021a; Chen et al., 2023a).

In addition to the GPX4-GSH axis, alternative ferroptosis defense pathways have been identified (Stockwell, 2022). FSP1 (ferroptosis suppressor protein 1) acts in parallel with GPX4, trapping lipid peroxy radicals by reducing coenzyme Q10 (CoQ10) to CoQ10H2 (Doll et al., 2019; Bersuker et al., 2019). DHODH (dihydroorotate dehydrogenase) provides mitochondrial CoQ10H2 as another defense layer (Mao et al., 2021). GTP cyclohydrolase 1 (GCH1) generates tetrahydrobiopterin (BH4), an endogenous radical-trapping antioxidant (Kraft et al., 2020). These parallel pathways provide redundant protection against ferroptosis and represent potential therapeutic targets.

### 2.2 Role of ferroptosis in fracture healing

Fracture healing is a highly ordered biological process involving inflammatory response, cartilaginous callus formation, and osseous callus remodeling stages, requiring precise coordination of multiple cell types (Einhorn and Gerstenfeld, 2015; Salhotra et al., 2020; Bebber et al., 2020). Ferroptosis regulates multiple aspects of fracture healing by affecting the survival and function of these key cells (Ru et al., 2023; Zhang et al., 2023a; Wang et al., 2024).

During the inflammatory stage, fractures lead to local hematoma formation and inflammatory cell infiltration, with sudden increases in pro-inflammatory cytokine release and ROS production (Einhorn and Gerstenfeld, 2015). A moderate inflammatory response is essential for clearing necrotic tissue and initiating the repair process, but excessive or persistent inflammation can impair fracture healing by inducing cellular ferroptosis (Bi et al., 2023; Yuan et al., 2024). *In vitro* studies show that osteoblasts in an inflammatory microenvironment exhibit ferroptosis characteristics, including decreased GPX4 expression, accumulation of lipid ROS, and decreased cell viability (Zhang et al., 2023a; Zhao et al., 2022). Treatment with ferroptosis inhibitors Ferrostatin-1 or Liproxstatin-1 significantly improves osteoblast survival and mineralization capacity under inflammatory conditions (Zilka et al., 2017; Zhang et al., 2024a).

During the callus formation stage, migration of mesenchymal stem cells (MSCs) to the fracture site and their differentiation into osteoblasts and chondrocytes is key to healing (Salhotra et al., 2020; Bebber et al., 2020). Studies show that ferroptosis can impair the proliferation, migration, and osteogenic differentiation capacity of MSCs (Yuan et al., 2024; Yang et al., 2024). Mechanistic research reveals that iron overload inhibits MSC osteogenic differentiation through activation of MAPK and NF-κB signaling pathways, and the iron chelator deferoxamine can reverse this effect (Zhang et al., 2023a; Gao et al., 2022). GPX4 overexpression can protect MSCs from oxidative stress-induced ferroptosis and promote expression of osteogenic-related genes (Runx2, Osterix, alkaline phosphatase) (Ruan et al., 2024; Lin et al., 2022).

Vascularization is critical for fracture healing, providing oxygen, nutrients, and osteoprogenitor cells to newly formed bone tissue (Einhorn and Gerstenfeld, 2015). Endothelial cells are highly sensitive to ferroptosis; iron overload and lipid peroxidation can impair endothelial cell function and inhibit angiogenesis (Fang et al., 2023). Studies show that vascular density at fracture sites is positively correlated with local GPX4 expression, and ferroptosis inhibitors can promote vascular regeneration and accelerate fracture healing (Bi et al., 2023; Wang et al., 2024).

### 2.3 Ferroptosis and postoperative muscle atrophy

Muscle atrophy is one of the most common and devastating complications after elderly hip fracture surgery, severely affecting functional recovery and quality of life (Handoll et al., 2021; Cruz-Jentoft et al., 2019). Traditional views hold that muscle atrophy mainly results from imbalance between decreased protein synthesis and increased degradation (Cruz-Jentoft et al., 2019; Dent et al., 2018). However, recent research has revealed the important role of ferroptosis in the pathogenesis of muscle atrophy (Huang et al., 2021; Ding et al., 2021).

Postoperative immobilization triggers metabolic and signaling pathway changes. Studies show that immobilization causes increased iron content in muscle tissue, elevated lipid peroxidation markers, and decreased GPX4 expression, suggesting ferroptosis participates in disuse muscle atrophy (Huang et al., 2021). Satellite cells (the muscle regeneration stem cell population) are highly sensitive to oxidative stress and ferroptosis; the decrease in satellite cell number and antioxidant capacity in elderly individuals increases ferroptosis sensitivity (Ding et al., 2021; Ahn et al., 2019; Chen et al., 2023b). Clinical studies have found that patients with low GPX4 expression in muscle biopsy specimens after hip fracture surgery have worse muscle strength recovery, suggesting GPX4 is a potential biomarker for predicting muscle rehabilitation (Huang et al., 2021; Cruz-Jentoft et al., 2019).

## 3 Role of lipid metabolism in the musculoskeletal system and its interaction with ferroptosis

### 3.1 Lipid metabolism and bone health

Lipids play multiple roles in bone metabolism as energy sources, signaling molecules, and membrane structural components (Rendina-Ruedy and Rosen, 2020). Abnormal fatty acid metabolism is closely associated with osteoporosis and increased fracture risk (Corrado et al., 2020). Studies show that excessive saturated fatty acid intake promotes inflammatory responses, inhibits osteoblast differentiation, enhances osteoclast activity, and leads to bone loss (Gao et al., 2022; Maheshwari et al., 2024). Conversely, ω-3 polyunsaturated fatty acids (EPA and DHA) have anti-inflammatory and pro-osteogenic effects, and ω-3 PUFA supplementation can improve bone mineral density and microstructure (Smith et al., 2015; Calder, 2017).

Lipid metabolism regulates the differentiation fate of bone marrow mesenchymal stem cells (BMSCs) (Rendina-Ruedy and Rosen, 2020; Li et al., 2016). BMSCs have the capacity to differentiate bidirectionally into osteoblasts and adipocytes, with differentiation balance regulated by multiple transcription factors and signaling pathways (Pino et al., 2012). PPAR-γ activation promotes adipogenesis while inhibiting osteogenic differentiation, whereas Runx2 and Wnt/β-catenin signaling promotes osteogenic differentiation (Meier et al., 2016; Rendina-Ruedy and Rosen, 2020; Zhang et al., 2024b). Elderly and osteoporotic individuals show increased bone marrow adipose tissue accompanied by decreased osteoblast generation; this shift toward adipocyte differentiation is associated with decreased bone mass and increased fracture risk (Corrado et al., 2020; Li et al., 2016).

### 3.2 Lipid metabolism and muscle function

Skeletal muscle is a highly metabolically active tissue, and lipid metabolism is essential for maintaining muscle function (Cruz-Jentoft et al., 2019; Conte et al., 2013). Fatty acid oxidation (FAO) is the primary energy source for muscle at rest and during low-intensity exercise. Decreased mitochondrial FAO capacity is associated with sarcopenia and muscle functional decline (Cruz-Jentoft et al., 2019; Chen et al., 2023b). Aging muscle exhibits mitochondrial dysfunction, including decreased mitochondrial density, reduced activity of respiratory chain complexes, and weakened fatty acid oxidation capacity, leading to insufficient energy supply and promoting muscle atrophy and strength decline (López-Otín et al., 2023; Ahn et al., 2019).

Intramuscular lipid accumulation (muscle fat infiltration) is a characteristic change in aging muscle, associated with impaired muscle function and metabolic disorders (Conte et al., 2013). Muscle membranes are rich in PUFAs, especially AA and DHA, which not only affect membrane fluidity and protein function but also generate biologically active lipid mediators (Smith et al., 2015; Dupont et al., 2019). ω-3 PUFA supplementation can improve muscle strength and function in the elderly (Calder, 2017; Dupont et al., 2019).

### 3.3 Lipid remodeling and ferroptosis sensitivity

**FIGURE 4:**
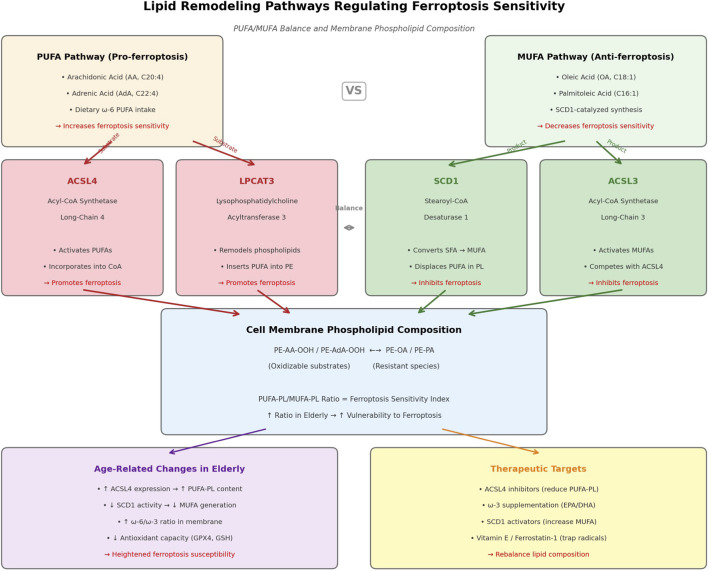
Lipid Remodeling Pathways Regulating Ferroptosis Sensitivity. The pro-ferroptotic (PUFA, left) and anti-ferroptotic (MUFA, right) pathways are shown. Key enzymes ACSL4 and LPCAT3 incorporate PUFAs into membrane phospholipids, increasing ferroptosis sensitivity; SCD1 and ACSL3 promote MUFA incorporation, conferring resistance. The PUFA–PL/MUFA–PL ratio determines the ferroptosis sensitivity index. Age–related changes (ACSL4↑, SCD1↓) tip the balance toward greater susceptibility.

Lipid composition, especially PUFA content, determines cellular sensitivity to ferroptosis (Liang et al., 2022; Pope and Dixon, 2023). PUFA-containing phospholipids (especially phosphatidylethanolamine/PE) are the primary substrates for lipid peroxidation (Kagan et al., 2017). Acyl-CoA synthetase long-chain family member 4 (ACSL4) selectively activates PUFAs, incorporating them into membrane phospholipids, thereby increasing ferroptosis sensitivity (Doll et al., 2017). Lysophosphatidylcholine acyltransferase 3 (LPCAT3) catalyzes PUFA incorporation into phospholipids, cooperating with ACSL4 to constitute the PUFA-driven ferroptosis pathway (Kagan et al., 2017). Conversely, incorporation of monounsaturated fatty acids (MUFAs) reduces ferroptosis sensitivity (Magtanong et al., 2019). Stearoyl-CoA desaturase 1 (SCD1) catalyzes conversion of saturated fatty acids to MUFAs, exhibiting anti-ferroptotic effects (Viswanathan et al., 2017). Elderly individuals show decreased SCD1 activity, with elevated membrane PUFA/MUFA ratio, potentially increasing ferroptosis susceptibility (Figure 4).

## 4 Ferroptosis mechanisms in tumor bone metastasis and pathological hip fractures

### 4.1 Molecular pathological basis of tumor bone metastasis

Tumor bone metastasis is one of the most common forms of distant metastasis of malignant tumors; in breast cancer, lung cancer, prostate cancer, renal cancer, and multiple myeloma patients, the incidence of bone metastasis can be as high as 60%–80% (Macedo et al., 2017). The process of tumor cell “homing” to the bone marrow depends on the chemokine axis (such as CXCL12-CXCR4), osteopontin, and integrin-mediated adhesion molecule interactions (Coleman et al., 2020). After colonization, tumor cells activate osteoclasts through paracrine mechanisms, secreting PTHrP, IL-6, TGF-β, and other factors to promote osteolytic destruction, forming a “vicious cycle”: bone matrix degradation releases TGF-β and other growth factors, further promoting tumor proliferation and bone destruction (Pei et al., 2022; Zhang et al., 2025).

The proximal femur, due to its abundant red marrow blood supply and hematopoietic microenvironment, provides ideal colonization conditions for tumor cells and is a high-incidence site for metastatic pathological fractures (Jiang et al., 2024). Compared with osteoporotic fractures, pathological fractures have higher and more prolonged local inflammatory cytokine concentrations, significantly lower fracture healing rates, and greater postoperative complication risk (Bebber et al., 2020).

### 4.2 Special influence of tumor microenvironment on ferroptosis regulation

The tumor microenvironment (TME) is a complex ecosystem composed of tumor cells, cancer-associated fibroblasts (CAFs), tumor-associated macrophages (TAMs), immune cells, and extracellular matrix, exerting multi-dimensional effects on ferroptosis regulation (Zhou et al., 2024). At the iron metabolism level, tumor cells exhibit “iron addiction”: high expression of TFR1 promotes iron uptake while downregulating FPN1 to impede iron export, leading to intracellular iron accumulation. Tumor-derived lipid carrier proteins (such as Lipocalin-2) can hijack iron from the bone marrow microenvironment, competitively depriving normal bone and muscle cells of iron supply (Bebber et al., 2020). Local iron overload in bone metastasis lesions simultaneously provides proliferative drive for tumor cells and causes iron toxicity damage to normal bone cells, activating excessive ferroptosis (Zhou et al., 2024).

Tumor-associated macrophages (TAMs) undergo M2 polarization in the bone metastasis microenvironment, releasing iron to tumor cells through iron cycling mechanisms, and upregulating GPX4 expression in tumor cells through STAT3 signaling, helping tumor cells escape ferroptosis (Liao et al., 2022). In contrast, bone marrow-derived mesenchymal stem cells and osteoblasts in the same environment are in a relatively ferroptosis-susceptible state, with GPX4 and FSP1 expression further suppressed by tumor-derived cytokines, exacerbating bone repair obstacles (Coleman et al., 2020).

Exosomes secreted by tumor cells can deliver pro-ferroptotic miRNAs (such as miR-9-5p) to normal cells, downregulating SLC7A11 expression in target cells and reducing GSH synthesis, significantly enhancing ferroptosis sensitivity of surrounding bone cells (Wang et al., 2021). This exosome-mediated ferroptosis “propagation” mechanism may be an important but underappreciated pathway for local bone destruction in tumor bone metastasis.

### 4.3 Special dilemmas of ferroptosis and fracture healing after pathological fractures

Surgical treatment of pathological fractures typically employs intramedullary nail fixation or arthroplasty; surgical intervention can restore mechanical stability but cannot change the tumor microenvironment’s continuous interference with bone healing (Jiang et al., 2024; Coleman et al., 2020). Residual tumor tissue continues to secrete inflammatory factors and pro-ferroptotic signals post-operatively, creating a “persistent ferroptosis induction state.” First, continuous activation of osteoblast ferroptosis: tumor-secreted DKK1 and Sclerostin inhibit Wnt/β-catenin signaling, while tumor-derived IL-1β downregulates SLC7A11, persistently undermining osteogenic capacity (Pei et al., 2022; Baracos et al., 2018). Second, impaired vascular regeneration: high concentrations of VEGF antagonists (such as Endostatin) in the TME combined with ferroptosis-induced endothelial cell damage jointly inhibit neovascularization at the fracture site (Bi et al., 2023; Coleman et al., 2020). Third, damage to the muscle-bone axis function: tumor cachexia (mediated by TNF-α, IL-6, GDF-15) overlaps with disuse atrophy, making muscle rehabilitation for pathological fracture patients face greater challenges (Cruz-Jentoft et al., 2019; Baracos et al., 2018).

### 4.4 Ferroptosis targeting strategies for pathological fractures

Given the unique nature of ferroptosis regulation in pathological fractures, intervention strategies need to achieve “selective ferroptosis regulation”—simultaneously inhibiting tumor cell proliferation while protecting normal bone cells (Zhou et al., 2024; Zhou et al., 2020; Guo et al., 2026). Research directions include: utilizing the GPX4 expression difference between tumor and normal cells to develop targeted agents; nanocarrier systems can achieve tumor microenvironment-responsive release of ferroptosis regulators (Yuan et al., 2024; Zhou et al., 2020); exosome-derived miRNA intervention to block tumor exosome interactions with osteoblasts to reduce ferroptosis “propagation” (Wang et al., 2021).

## 5 Genetic and epigenetic regulation of ferroptosis and lipid metabolism

### 5.1 Genetic polymorphisms of key genes

Genetic polymorphisms of ferroptosis-related pathways play important roles in inter-individual differences in ferroptosis sensitivity, providing genetic basis for understanding individual differences in elderly fracture rehabilitation trajectories (Zhou et al., 2023; Shi et al., 2022).

Single nucleotide polymorphisms (SNPs) of the GPX4 gene (located at 19p13.3) affect its protein expression level and enzymatic activity. Studies have found that GPX4 rs713041 (C/T) polymorphism is significantly associated with oxidative stress sensitivity; T allele carriers show reduced GPX4 mRNA stability and are more susceptible to ferroptosis under stress conditions (Pei et al., 2022). The SLC7A11 gene encodes the core subunit xCT of system xc^−^, and SNPs in its promoter region affect transcription factor binding efficiency and gene expression levels. SLC7A11 low-expression variants are associated with insufficient GSH synthesis and increased ferroptosis susceptibility; simultaneously, SLC7A11 tends to be overexpressed in tumors to help tumor cells escape ferroptosis (Tang et al., 2021; Zhou et al., 2024).

ACSL4 gene (located at Xq22.3-q23) polymorphisms affect PUFA incorporation efficiency and ferroptosis substrate supply. The ACSL4 rs340811 polymorphism is associated with lipid peroxidation levels and muscle functional decline (Doll et al., 2017; Shi et al., 2022). Additionally, genetic variants of iron metabolism-related genes (HFE, HAMP, TFR2) affect systemic iron homeostasis; HFE gene C282Y and H63D mutations are pathogenic variants of hereditary hemochromatosis, and their heterozygous carriers are more prone to iron overload under stress conditions, theoretically increasing post-fracture ferroptosis risk (Chen et al., 2020; Feder et al., 1996).

### 5.2 Epigenetic modifications regulating ferroptosis pathways

DNA methylation, histone modifications, and non-coding RNAs constitute the epigenetic network of ferroptosis regulation, and undergo systematic reprogramming with aging (Pei et al., 2022; Ye et al., 2021). DNA methylation has important regulatory effects on GPX4 and SLC7A11 expression. Studies have found that hypermethylation of GPX4 promoter CpG islands in osteoblasts of elderly osteoporotic patients is associated with GPX4 transcriptional suppression, and DNMT-mediated epigenetic silencing can accelerate osteoblast ferroptosis and promote osteoporosis progression (Ruan et al., 2024). Importantly, DNMT inhibitor (such as 5-azacytidine) treatment can restore GPX4 expression, suggesting that epigenetic editing is a potential intervention strategy.

Non-coding RNAs play key roles in ferroptosis regulation. miR-21-5p reduces SLC7A11 expression by targeting its 3′UTR, increasing cellular ferroptosis sensitivity; overexpression of miR-21-5p in elderly skeletal muscle is associated with GPX4 pathway inhibition and impaired satellite cell regeneration (Ding et al., 2021). lncRNA GPRC5D-AS1 promotes SLC7A11 expression in skeletal muscle cells, exerting ferroptosis protection; its downregulation in sarcopenia is associated with enhanced ferroptotic cell death, which may similarly compromise osteoblast survival under iron-overload conditions (Gong et al., 2025). Circular RNA circFADS2, by binding fatty acid desaturase 2 (FADS2), regulates PUFA generation, affecting ferroptosis substrate supply; in muscle atrophy models, downregulation of circFADS2 expression is associated with ferroptosis activation (Ding et al., 2021).

### 5.3 Genetic and epigenetic regulation of lipid metabolism-related genes

FADS1/FADS2 (fatty acid desaturase gene cluster) polymorphisms affect the efficiency of PUFA endogenous synthesis; the FADS1 rs174537 polymorphism is associated with plasma arachidonic acid (AA) levels and ferroptosis substrate supply (Tanaka et al., 2009). APOE gene polymorphisms (ε2/ε3/ε4) affect lipid transport and cholesterol metabolism; the APOE ε4 allele is associated with increased lipid peroxidation susceptibility (Corrado et al., 2020). During aging, hypomethylation of the PPAR-γ promoter in bone marrow adipocytes promotes BMSC differentiation toward adipocytes, reducing osteoblast derivation and forming a link to increased osteoporotic fracture risk (Li et al., 2016; Doudna and Charpentier, 2014).

### 5.4 Potential for genomics-driven precision intervention

Multi-omics integrated analysis provides new dimensions for ferroptosis-related precision medicine (Tabassum et al., 2021; Collins and Varmus, 2015). By integrating genomic (SNPs), epigenomic (methylation), transcriptomic (mRNA/ncRNA), and lipidomic data, multi-level ferroptosis sensitivity assessment models can be constructed to guide individualized intervention strategies (Pei et al., 2022; Shi et al., 2022). Gene editing technologies represented by CRISPR-Cas9 provide powerful tools for exploring ferroptosis-related gene functions and provide a theoretical basis for future gene therapy (Doudna and Charpentier, 2014). With the maturation of single-cell sequencing technology, resolving ferroptosis-related gene expression profiles at the cell subpopulation level will provide unprecedented resolution for understanding the ferroptosis status of different cell types in the fracture repair microenvironment (Ruan et al., 2024; Jiang et al., 2024).

## 6 Nursing assessment, intervention, and multidisciplinary rehabilitation management

### 6.1 Nursing assessment system for elderly hip fractures

Systematic nursing assessment is the starting point for developing precision rehabilitation plans, encompassing physiological, psychological, social functional, and molecular biomarker dimensions (Handoll et al., 2021; Avenell et al., 2016). Functional status assessment should use: the Barthel Index (BI), Harris Hip Score (HHS), Short Physical Performance Battery (SPPB), and handgrip strength (Cruz-Jentoft et al., 2019; Avenell et al., 2016). Dynamic monitoring at various time points after surgery can accurately delineate functional recovery trajectories.

Nutritional risk screening is a key component: Mini Nutritional Assessment (MNA) or Nutritional Risk Screening 2002 (NRS 2002) should be completed within 24 h of admission (Dent et al., 2018; Avenell et al., 2016). For patients with tumor pathological fractures, tumor cachexia assessment (GLIM criteria) should be incorporated to distinguish simple malnutrition from cachexia (Baracos et al., 2018). Oxidative stress and iron metabolism biomarker monitoring recommended includes: serum ferritin, transferrin saturation (TSAT), plasma MDA and 4-HNE concentrations, serum GPX activity, and plasma F2-isoprostanes (Rochette et al., 2023; Ayala et al., 2014; Xie et al., 2023; Zhou et al., 2023).

Pain assessment should adopt a multimodal strategy using NRS/VAS scales, with special attention to tumor-related pain in pathological fracture patients. Fall risk assessment (Morse fall Scale), pressure injury risk (Braden Scale), cognitive and psychological assessment (MMSE, GDS, HADS) are essential components of elderly fracture nursing (Handoll et al., 2021; Fairhall et al., 2022; Avenell et al., 2016).

### 6.2 Early postoperative nursing interventions

Early postoperative nursing interventions (24–72 h after surgery) are critical for preventing complications and initiating functional recovery (Handoll et al., 2021; Fairhall et al., 2022). Positioning management and early mobilization are core components. Patients are encouraged to begin active bed exercises within 24 h after surgery (ankle pump exercises, quadriceps isometric contractions, straight leg raises) to prevent DVT and muscle atrophy (Fairhall et al., 2022; Avenell et al., 2016). From a ferroptosis regulation perspective, early mobilization activates PGC-1α signaling to enhance mitochondrial biogenesis and FAO capacity, upregulates the NRF2-SLC7A11-GPX4 axis, and suppresses muscle ferroptosis (Huang et al., 2021; Dodson et al., 2019).

Pain management should adopt a multimodal analgesia (MMA) regimen: NSAIDs or acetaminophen as baseline, combined with regional nerve blocks (such as fascia iliaca compartment block/FICB) to reduce opioid use (Handoll et al., 2021; Avenell et al., 2016). Nutritional intervention should begin in the early postoperative period; for cachectic patients, collaborate with dietitians to develop high-protein (≥1.5 g/kg/d) antioxidant nutrient-enhanced regimens (Dent et al., 2018; Bauer et al., 2013).

### 6.3 Systematic nursing intervention strategies during the rehabilitation period

Nutritional intervention should continue and be dynamically adjusted throughout the rehabilitation period. Protein supplementation (1.2–1.5 g/kg body weight/day) is the basis for maintaining muscle anabolic metabolism; whey protein rich in leucine is the preferred supplementation form (Cruz-Jentoft et al., 2019; Dent et al., 2018; Bauer et al., 2013). ω-3 fatty acid supplementation (EPA + DHA 2–4 g/day) promotes muscle recovery and reduces ferroptosis substrate by exerting anti-inflammatory effects and improving membrane phospholipid PUFA/MUFA ratio (Smith et al., 2015; Calder, 2017; Dupont et al., 2019).

Antioxidant micronutrients: Vitamin E (α-tocopherol, 400–800 IU/day) directly blocks membrane phospholipid peroxidation chain reactions; selenium (40–70 μg/day) is an essential element for GPX4 catalytic activity, and moderate supplementation enhances GPX4 antioxidant capacity (Xie et al., 2023; Sies et al., 2022). Vitamin D (800–2000 IU/day) regulates calcium and phosphorus metabolism and indirectly reduces the ferroptosis-inducing environment (Bischoff-Ferrari et al., 2016). Iron nutritional status needs individualized assessment; recommended target serum ferritin range is 50–200 μg/L (Chen et al., 2020; Brissot et al., 2020; Avenell et al., 2016).

Exercise rehabilitation (Progressive Resistance Training/PRT) promotes muscle protein synthesis via mTOR-S6K1 pathway, improves mitochondrial function via AMPK-PGC-1α pathway, and upregulates NRF2-mediated antioxidant defense; additionally, resistance exercise can upregulate muscle GPX4 expression, directly enhancing ferroptosis defense capacity (Huang et al., 2021; Ding et al., 2021).

### 6.4 Psychological nursing and social support

Psychological factors have profound effects on fracture rehabilitation and have a bidirectional relationship with the ferroptosis-inflammation axis. Depressive symptoms activate the HPA axis to elevate glucocorticoid levels, promoting muscle catabolism, while simultaneously exacerbating oxidative stress and ferroptosis sensitivity by increasing pro-inflammatory cytokines (IL-6, TNF-α) (Handoll et al., 2021; Avenell et al., 2016). Studies show that the incidence of depression in elderly hip fracture patients reaches 20%–40%. Nursing staff should regularly implement GDS assessment and adopt cognitive-behavioral support interventions (Handoll et al., 2021).

### 6.5 Transitional care and post-discharge management

Transitional care ensures that inpatient rehabilitation benefits extend to community and home settings (Handoll et al., 2021; Fairhall et al., 2022). Regular follow-up monitoring should include: functional assessment (Harris Hip Score, gait speed, handgrip strength); imaging assessment (fracture healing progress); biochemical monitoring (serum ferritin, GPX activity, MDA, albumin); fall risk re-assessment (Zhou et al., 2023; Sànchez-Rodríguez et al., 2020). Application of telehealth technology and wearable devices provides continuous monitoring and support for home rehabilitation patients (Collins and Varmus, 2015).

### 6.6 Multidisciplinary collaborative nursing model

Optimal nursing of elderly hip fractures relies on a patient-centered multidisciplinary collaborative model (MDT), with nursing staff playing a coordinating hub role (Handoll et al., 2021; Avenell et al., 2016). The Orthogeriatric Co-management model has been confirmed by multiple systematic reviews to reduce in-hospital mortality, shorten hospital stay, and reduce complications (Handoll et al., 2021). Nurse-led case management coordinates specialized resources from orthopedic surgery, geriatrics, nutrition, physical therapy, occupational therapy, social work, and psychology. For pathological fracture patients, the inclusion of oncology and palliative care specialties further expands MDT composition (Baracos et al., 2018; Avenell et al., 2016).

## 7 From mechanisms to clinical translation

### 7.1 Biomarker discovery and validation

Translating basic research findings on ferroptosis and lipid metabolism into clinical applications first requires identifying reliable biomarkers for risk stratification, efficacy monitoring, and prognostic prediction (Zhou et al., 2023; Tabassum et al., 2021). Serum/plasma biomarkers for ferroptosis include: ferritin and soluble transferrin receptor reflecting systemic iron status (Chen et al., 2020; Brissot et al., 2020); lipid peroxidation products (MDA, 4-HNE, F2-isoprostanes) indicating oxidative stress levels (Rochette et al., 2023; Ayala et al., 2014); GPX4 and GPX activity reflecting antioxidant defense capacity (Xie et al., 2023). Lipidomics provides powerful tools for comprehensive evaluation of lipid metabolism, simultaneously quantifying hundreds of lipid molecules through mass spectrometry (Tabassum et al., 2021). For pathological fracture patients, liquid biopsy (ctDNA, tumor-derived exosomes) can simultaneously provide tumor progression information and ferroptosis regulation status information (Zhou et al., 2024; Wang et al., 2021).

### 7.2 Pharmacological intervention strategies

Iron chelators (deferoxamine/DFO, deferiprone/DFP, deferasirox/DFX) prevent ferroptosis by binding free iron and reducing Fenton reactions (Kontoghiorghes et al., 2020). DFO pre-treatment can alleviate immobilization-induced muscle atrophy and improve fracture healing (Zhang et al., 2023a; Yang et al., 2024). Antioxidants including N-acetylcysteine (NAC) as a glutathione precursor can replenish intracellular GSH and enhance GPX4 activity (Zheng and Conrad, 2020). Specific ferroptosis inhibitors Ferrostatin-1 and Liproxstatin-1 block ferroptosis by trapping lipid peroxy radicals (Zilka et al., 2017). ω-3 polyunsaturated fatty acid supplementation has shown skeletal and muscle protective effects in multiple studies (Smith et al., 2015; Calder, 2017; Dupont et al., 2019). Selective ACSL4 inhibitors and SCD1 activators are under development as lipid metabolism regulators (Doll et al., 2017; Magtanong et al., 2019; Viswanathan et al., 2017).

### 7.3 Nutritional intervention strategies

Nutritional intervention is an important strategy for improving rehabilitation in elderly hip fracture patients (Dent et al., 2018; Avenell et al., 2016). Protein supplementation (1.2–1.5 g/kg body weight/day) is critical for combating muscle atrophy (Cruz-Jentoft et al., 2019; Dent et al., 2018; Bauer et al., 2013). ω-3 fatty acid supplementation (2–4 g EPA + DHA/day) provides anti-inflammatory and metabolic benefits (Calder, 2017; Dupont et al., 2019). Antioxidant nutrient supplementation (vitamins E and C, selenium) can enhance antioxidant defense and reduce oxidative stress (Sies et al., 2022). Vitamin D (800–2000 IU/day) regulates calcium and phosphorus metabolism and has immunomodulatory and muscle function-improving effects (Bischoff-Ferrari et al., 2016). Iron nutritional status requires individualized assessment by monitoring serum ferritin, transferrin saturation, and hemoglobin levels (Avenell et al., 2016).

### 7.4 Precision medicine and individualized rehabilitation

**FIGURE 5:**
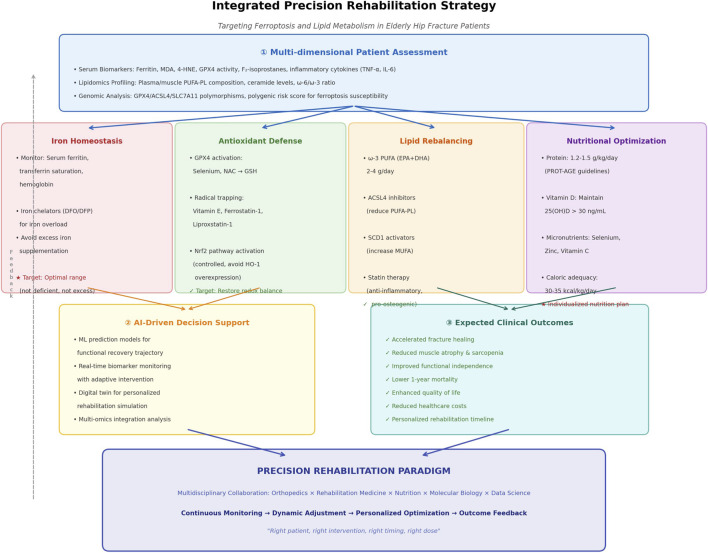
Integrated Precision Rehabilitation Strategy Targeting Ferroptosis, Lipid Metabolism, and Gene Regulation in Elderly Hip Fracture Patients. The framework begins with multi-dimensional patient assessment (biomarkers, lipidomics, genomics), flows through four intervention pillars: iron homeostasis management, antioxidant defense enhancement, lipid metabolism re-balancing, and nutritional/exercise optimization. AI-driven decision support enables real-time adaptive interventions, leading to improved clinical outcomes. The precision rehabilitation paradigm emphasizes multidisciplinary collaboration and continuous feedback optimization.

Precision medicine emphasizes precise disease classification and individualized treatment based on individual multi-omics information (Collins and Varmus, 2015). Applying precision medicine approaches to elderly hip fracture rehabilitation can identify high-risk patients, predict rehabilitation trajectories, and optimize intervention strategies (Rajkomar et al., 2019; Farrow et al., 2024). GPX4 gene variants are associated with oxidative stress sensitivity; ACSL4 and LPCAT3 gene polymorphisms affect lipid remodeling and ferroptosis susceptibility (Doll et al., 2017; Kagan et al., 2017). Multi-omics integrated analysis provides a systemic perspective (Tabassum et al., 2021; Collins and Varmus, 2015). Using machine learning algorithms to build functional recovery prediction models after hip fracture surgery can guide individualized rehabilitation plan development (Rajkomar et al., 2019; Farrow et al., 2024) (Figure 5).

## 8 Challenges, future directions, and prospects

### 8.1 Current research limitations

Despite significant progress, translating basic findings into clinical applications still faces many challenges (Stockwell, 2022; Berndt et al., 2024). Most ferroptosis research is based on *in vitro* cell experiments and animal models, with limited validation in human tissues (Yan et al., 2021). Elderly-specific pathophysiological features and tumor microenvironments are difficult to fully reproduce in existing models (López-Otín et al., 2023; Bebber et al., 2020). Methodologies for detecting ferroptosis and lipid metabolism are not yet complete; lipid peroxidation products have short half-lives and low methodological standardization (Rochette et al., 2023; Zhou et al., 2023). Causal relationships between ferroptosis/lipid metabolism and fracture rehabilitation remain unclear, requiring well-designed prospective cohort studies and interventional clinical trials (Ru et al., 2023; Bi et al., 2023; Wang et al., 2024; Zhang et al., 2024b). For pathological fractures, the complexity of interactions between tumor type heterogeneity, diverse tumor treatment regimens, and ferroptosis regulation makes study design more difficult. Studies associating genetic polymorphisms with ferroptosis susceptibility generally have insufficient sample sizes, lacking large-scale genomic studies in elderly fracture populations (Shi et al., 2022).

### 8.2 Future research directions

At the mechanistic research level: using single-cell sequencing technology to resolve ferroptosis-related gene expression profiles at the cell subpopulation level; deeply exploring the crosstalk between ferroptosis and other cell death modes (apoptosis, pyroptosis, autophagy); revealing the spatial heterogeneity of ferroptosis in the tumor microenvironment (Ruan et al., 2024; Jiang et al., 2024; Zhou et al., 2024). At the biomarker research level: establishing multi-center fracture patient biobanks for systematic validation studies; incorporating liquid biopsy technology into pathological fracture monitoring systems (Zhou et al., 2023; Tabassum et al., 2021; Wang et al., 2021). At the therapeutic research level: developing GPX4 activators, ACSL4 inhibitors, and FSP1 activators with good bioavailability; constructing tumor microenvironment-responsive nanodrug delivery systems; exploring CRISPR-mediated epigenetic editing in restoring ferroptosis defense (Lei et al., 2022; Doll et al., 2019; Zhang et al., 2024a; Doudna and Charpentier, 2014).

At the nursing and precision rehabilitation research level: conducting randomized controlled trials of ferroptosis biomarker-guided nursing interventions; utilizing wearable devices and digital health technologies for continuous home rehabilitation monitoring; constructing elderly fracture precision rehabilitation prediction platforms integrating multi-omics data, nursing assessment, and functional outcomes (Collins and Varmus, 2015; Rajkomar et al., 2019; Farrow et al., 2024; Sànchez-Rodríguez et al., 2020). Artificial intelligence and machine learning have broad application prospects; deep learning algorithms can extract features from multimodal data to build prediction models (Rajkomar et al., 2019).

### 8.3 Multidisciplinary collaboration

Translating research results into clinical practice requires close multidisciplinary collaboration (Handoll et al., 2021; Collins and Varmus, 2015). Clinical trial design requires innovation including adaptive designs and platform trials (Collins and Varmus, 2015). Patient participation is crucial; shared decision-making respects patient autonomy (Handoll et al., 2021). Health education and public participation increase awareness of osteoporosis and fracture prevention (Wu et al., 2021; Sing et al., 2023; Yan et al., 2022). Perioperative infection management, including fungal pathogens that exploit metabolic adaptation to evade host defenses, represents an additional dimension of multidisciplinary care for immunocompromised elderly fracture patients (Lok et al., 2026). The nursing discipline should actively participate in the translational process of ferroptosis-lipid metabolism research, integrating molecular biology findings into nursing assessment and intervention frameworks, and promoting precision development of the nursing discipline.

## 9 Conclusion

Ferroptosis and lipid metabolism play pivotal roles in postoperative rehabilitation of elderly hip fractures (including tumor metastatic pathological fractures), providing new perspectives for understanding the molecular mechanisms of the rehabilitation process (Jiang et al., 2021; Stockwell, 2022; Ru et al., 2023). Ferroptosis impairs fracture healing and muscle recovery through lipid peroxidation, iron overload, and antioxidant defense imbalance (Huang et al., 2021; Zhang et al., 2023a); lipid metabolism abnormalities not only increase ferroptosis sensitivity but also directly affect osteocyte and myocyte function (Liang et al., 2022; Pope and Dixon, 2023; Doll et al., 2017). In the context of tumor bone metastasis, the tumor microenvironment further amplifies the above dysregulation through iron metabolism hijacking, exosome-mediated epigenetic manipulation, and secretion of pro-ferroptotic factors (Coleman et al., 2020; Pei et al., 2022; Bebber et al., 2020; Zhou et al., 2024). Genetic polymorphisms and epigenetic modifications (DNA methylation, ncRNA regulation) play decisive roles in individual differences in ferroptosis sensitivity, providing genetic basis for precision rehabilitation stratification (Pei et al., 2022; Shi et al., 2022; Ye et al., 2021).

A multi-dimensional, integrated nursing assessment and intervention system—encompassing oxidative stress biomarker monitoring, precision nutritional support (protein, ω-3 fatty acids, selenium, vitamin D, vitamin E), early exercise rehabilitation, and psychosocial support—can not only improve functional outcomes but also exert regulatory effects on the ferroptosis-lipid metabolism axis at the molecular level (Handoll et al., 2021; Dent et al., 2018; Avenell et al., 2016).

Under the guidance of precision medicine, integrating multi-omics data to develop individualized plans represents the future direction (Collins and Varmus, 2015; Rajkomar et al., 2019; Farrow et al., 2024). Although challenges remain, ferroptosis, lipid metabolism, and genetic regulation research brings new hope for elderly hip fracture rehabilitation. As mechanistic understanding deepens and multidisciplinary collaboration strengthens, precision rehabilitation strategies are expected to enter clinical practice and significantly improve functional recovery and quality of life in elderly patients (Sing et al., 2023; Handoll et al., 2021; Berndt et al., 2024).
